# Supplementary material for: The causal relationship between the human gut microbiota and pyogenic arthritis: a Mendelian randomization study
Source: Front Cell Infect Microbiol. 2024 Nov 26;14:1452480. doi: 10.3389/fcimb.2024.1452480 (PMC11629706; doi:10.3389/fcimb.2024.1452480)
Supplement: Supplementary file 1 [file DataSheet1.zip › mendelian test/ebi-a-GCST90027633.csv_forest.pdf]

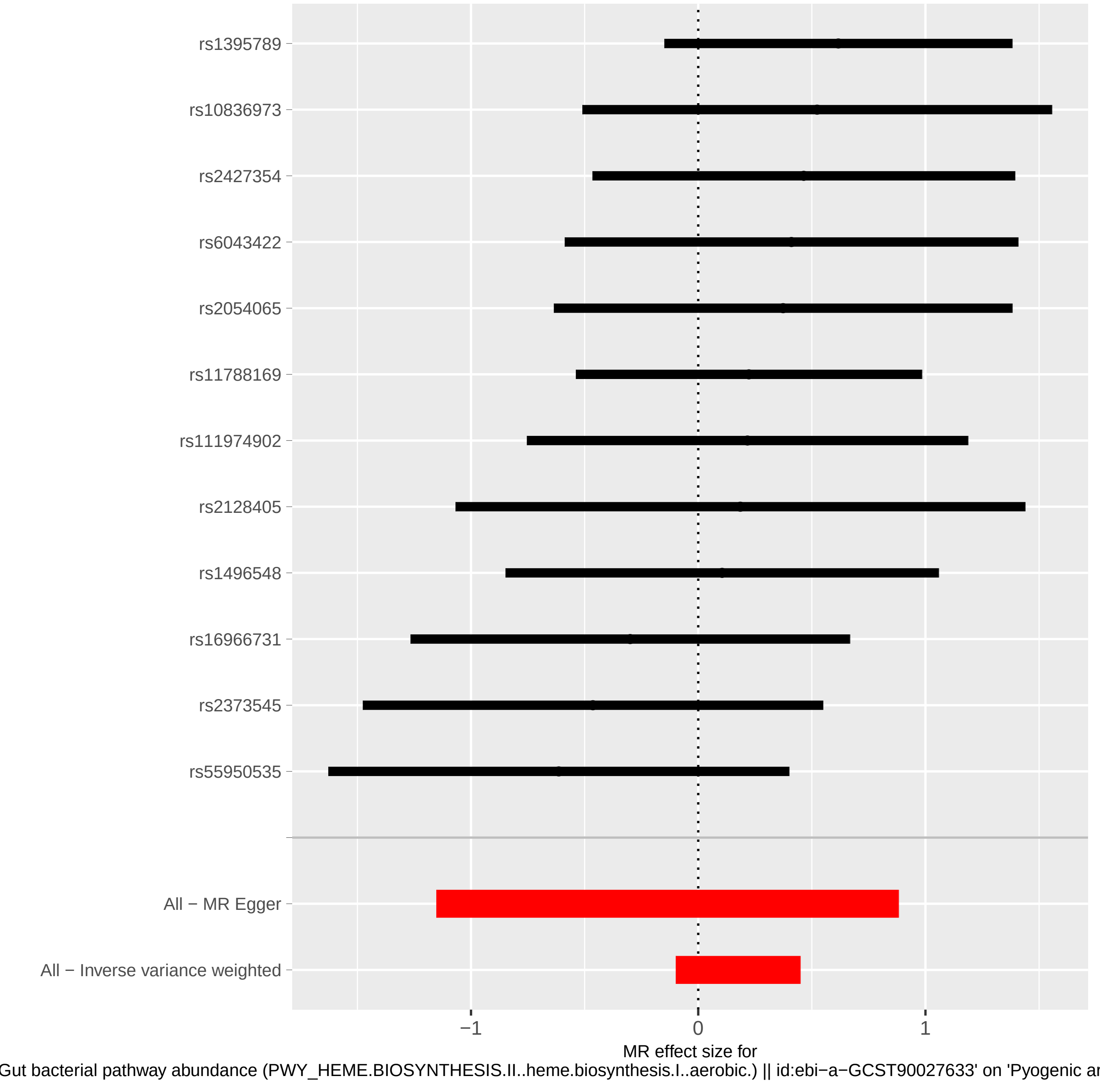
rs1395789
rs10836973
rs2427354
rs6043422
rs2054065
rs11788169
rs111974902
rs2128405
rs1496548
rs16966731
rs2373545
rs55950535
All − MR Egger
All − Inverse variance weighted
−1
0
1
MR effect size for
'Gut bacterial pathway abundance (PWY_HEME.BIOSYNTHESIS.II..heme.biosynthesis.I..aerobic.) || id:ebi−a−GCST90027633' on 'Pyogenic ar
